# Detection of Diarrheic Shellfish Poisoning and Azaspiracid Toxins in Moroccan Mussels: Comparison of the LC-MS Method with the Commercial Immunoassay Kit

**DOI:** 10.3390/md6040587

**Published:** 2008-12

**Authors:** Adra Elgarch, Paulo Vale, Saida Rifai, Aziz Fassouane

**Affiliations:** 1 Laboratoire de Biochimie, Faculté des Sciences, Université Chouaib Doukkali El jadida, Marocco; 2 IPIMAR, Instituto Nacional de Investigação Agrária e das Pescas, Av. Brasíilia, 1449-006- Lisboa, Portugal

**Keywords:** Diarrheic shellfish poisoning, Okadaic acid, LC/MS, ELISA, Dinophysistoxin 2, *Dinophysis spp.*, azaspiracids toxins

## Abstract

Diarrheic shellfish poisoning (DSP) is a recurrent gastrointestinal illness in Morocco, resulting from consumption of contaminated shellfish. In order to develop a rapid and reliable technique for toxins detection, we have compared the results obtained by a commercial immunoassay-“DSP-Check” kit” with those obtained by LC-MS. Both techniques are capable of detecting the toxins in the whole flesh extract which was subjected to prior alkaline hydrolysis in order to detect simultaneously the esterified and non esterified toxin forms. The LC-MS method was found to be able to detect a high level of okadaic acid (OA), low level of dinophysistoxin-2 (DTX2), and surprisingly, traces of azaspiracids 2 (AZA2) in mussels. This is the first report of a survey carried out for azaspiracid (AZP) contamination of shellfish harvested in the coastal areas of Morocco. The “DSP-Check” kit was found to detect quantitatively DSP toxins in all contaminated samples containing only OA, provided that the parent toxins were within the range of detection and was not in an ester form. A good correlation was observed between the two methods when appropriate dilutions were performed. The immunoassay kit appeared to be more sensitive, specific and faster than LC-MS for determination of DSP in total shellfish extract.

## 1. Introduction

Diarrheic shellfish poisoning is a severe gastrointestinal illness caused by consumption of seafood contaminated with toxigenic dinoflagellates such as certain species of the genus *Dinophysis* and *Prorocentrum* algae. The European Commission has subdivided DSP monitoring into four distinct families: dinophysistoxins (OA, DTX1, DTX2, and DTX3) and pectenotoxins (PTX1 and PTX2) with a maximum limit of 160 μg/kg, yessotoxins (YTXs) at a maximum limit 1 mg/kg level and azaspiracids (AZA1-3) at a maximum limit of 160 μg/kg shellfish meat.

Highly sensitive methods are required to detect DSP toxins at low concentrations. The HPLC method used by Lee *et al.* [1], despite using the highly fluorescent reagent 9-anthryldiazomethane (ADAM), is not very sensitive for detecting toxins at very low levels because of the chemical noise background. It is also laborious, time-consuming and, in practice, duplicate or triplicate analyses are carried out in the experiment. Additionally, an alkaline hydrolysis necessary for quantifying simultaneously OA and its ester derivatives in monitoring analyses, recently proposed by several authors [2] was found to increase the time of sample preparation. Consequently, several biochemical (phosphatase inhibition assays and enzyme linked immunosorbent assays) and biological (tissue culture assays) methods for detecting DSP toxins with a higher sample throughput have been proposed [3]. Interestingly, antibodies against DSP toxins have been developed only against okadaic acid.

So far, the diarrheic shellfish poisoning parent toxins found in Moroccan bivalves are OA and DTX2. The first detection of these toxins in the Mediterranean coast of Morocco was in oysters and clams from the Nador area in 1999 and in mussels in 2003. On the Atlantic coast, the first detection was in clams, also in 1999, and then mussel and oyster samples in 2000 and 2002. In 2003, this type of contamination was very important, and DSP was detected in mussel, clam and oyster samples along the Atlantic litoral from El Jadida to Dakhla.

Currently, a mouse bioassay is used in the Moroccan monitoring program. However, the introduction of a rapid, selective and quantitative assay is very important for proper risk management of this recurrent toxicity. Now, we report the detection of DSP toxins in mussels collected in Oualidia lagoon by using two methods: a commercial enzyme-linked immunoabsorbent assay (ELISA) [4], and liquid chromatography-mass spectrometry (LC-MS). Both techniques employed the same whole fresh final extract, subjected to prior alkaline hydrolysis in order to detect simultaneously the esterified and non-esterified toxin forms [5]. The “DSP-check” kit was then compared with the LC-MS method for determining its predicting capabilities for the complex toxin profiles found in Moroccan shellfish.

## 2. Results and Discussion

### 2.1. Detection of DSP and AZP toxins by LC-MS

**Figure 1.**
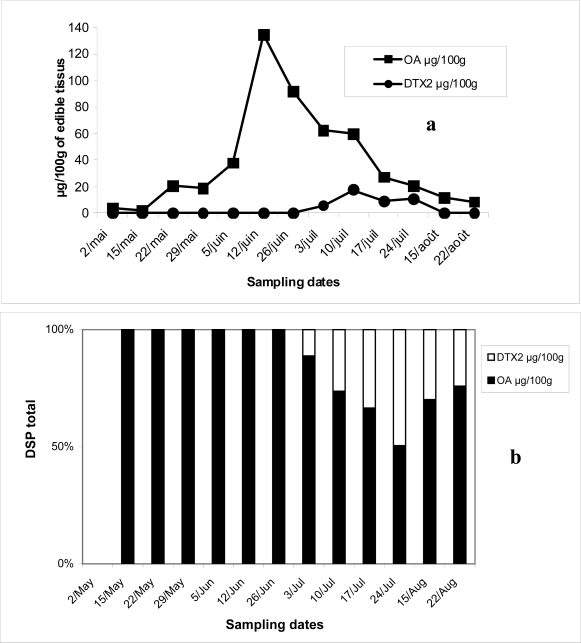
Evolution of OA and DTX2 (μg/100g of edible tissues) in mussels from Oualidia lagoon harvested between May and August 2006 (a) and their respective percentages (b).

Figure 1a shows the contamination of DSP in the mussels with both OA and DTX2. OA was present in high concentrations, 19 to 135 μg/100g, from May to August 2006, exceeding the public health safety threshold of 16 μg/100 g of edible tissues. The contamination depended on the period of collection and the highest level of DSP was registered in June, while the lowest level was found in May. During the toxic season, the percentage of DTX2 was from 9 to 23 % of total DSP toxins (Figure 1b). However, the level registered did not exceed the safety threshold.

**Figure 2.**
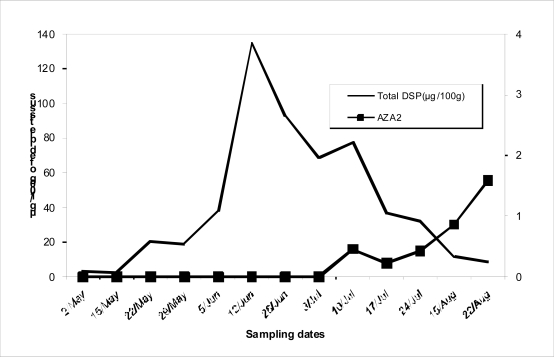
Evolution of total DSP and AZA2 in mussels from Oualidia lagoon harvested between May and August 2006.

Analysis carried out in the SIM mode for AZAs in mussels collected in the Oualidia lagoon in Morocco showed the presence, in some samples, of AZA 2 during July and August (Figure 2).

### 2.2. Detection of DSP toxins by ELISA assay

According to the sample preparation scheme, the detection limit of the ELISA method is 2.5 μg/100 g and 0.4 μg/100g for LC-MS, respectively. Thus, only samples that gave the results above the ELISA detection limit were chosen to compare quantitatively with those obtained by LC-MS. The results of comparison between ELISA and LC-MS are presented in Table 1. However, the values obtained by ELISA were similar to those obtained for OA content by the method of Lee *et al.* [1]. As the antibody is specific for OA, samples containing DTX2 showed a consistent tendency to present a higher DSP content by LC-MS than by ELISA.

The results obtained from the samples containing DSP toxins from the Oualidia lagoon in 2006 revealed a high level of OA in mussels. The highest concentration peak of OA was observed in the samples collected in June. Interestingly, the samples collected in July showed not only an increase of DSP toxins, but also the high levels of both OA and DTX2. Unfortunately we have no identification of *Dinophysis*. In Portuguese mussels *Dinophysis acuminata* has been found to be responsible only for the OA contamination, while *Dinophysis acuta* is responsible for both OA and DTX2 contamination [6,7]. The DSP peak observed in June could be caused by blooming of *D. acuminata*, while in July *D. acuta* growth could have been responsible by DTX2.

During this investigation, AZP toxins were detected for the second time in Moroccan mussels, in Oualidia lagoon area, from 13 samples of mussels, in five consecutive samples harvested from July to August apparently contained traces of AZA2, at levels up 6μg/kg, never surpassing the current EU limit.

The presence AZA2 as the dominant form of the azaspiracid family in mussels collected in the Atlantic coast of Morocco was reported for the first time during the summer of 2004 and 2005 [8] So far, AZP has been found only in northern European costs such as Ireland, England, Norway, and France as well as in Galicia [9,10] and in Northwest coast of Portugal [7] and the suspected producers of the toxins are the microalgae *Protoperidinium crassipes* [11,12], belonging to a large and ubiquitous phytoplankton genus. The risk of human outbreaks of AZP seems to be very low, compared with amnesic shellfish poisoning (ASP), or to diarrheic shellfish poisoning and paralytic shellfish poisoning (PSP). Taking into account the limits currently in force in the European Union, the AZP risk seems much lower than the ASP risk, but azasperacid induces adverse effects in mice after orally administered sublethal doses [13].

The LC-MS used to identify DSP toxins in Moroccan mussels, has detected OA and DTX2. These data were compared with ELISA assays. The “DSP-Check” kit was capable of detecting quantitatively DSP toxins in the entire contaminated samples tested within the detection range and were not in an ester form.

**Figure 3.**
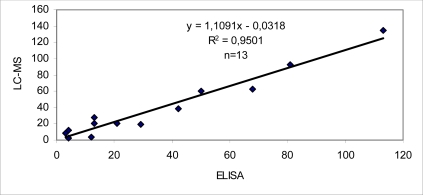
Correlation between ELISA and LC-MS .The results are grouped according to the samples containing OA (μg/100g edible tissues). (n= number of samples containing OA).

A high correlation was observed between ELISA and HPLC (Figure 3). The kit has a short linear range (1 order of magnitude: 10 to 100 ng/mL) when compared to LC-MS (two orders of magnitude: 1–80 ng/injection). This is not disadvantageous for public health protection, which was the main objective of this work. In fact, ELISA is more sensitive and faster than HPLC for determination of DSP in total meat extracts. HPLC is more valuable for research purposes because it has a superior linear range and can determine toxin profiles, which vary in accordance with the plankton available as a food source [14]. Currently, the mouse bioassay is still used in the Moroccan monitoring and control program (Institut National de Recherche Halieutique) as the official method for detecting biotoxins in shellfish. As the high DSP toxicity in the mouse bioassay of some Moroccan samples of mussels was probably regarded as being caused by other toxins, e.g. yessotoxins, pectenotoxins and azaspiracids, it is desirable to use the method that can detect them. So, we recommended the use the LC-MS in the Moroccan monitoring for a proper risk management of this recurrent toxicity.

## 3. Experimental

### 3.1. Sample preparation

Extractions were carried out according to the slightly modified method of Lee *et al.* [1] briefly, 80% aqueous methanol (20 mL) was added to 50 mL screw-cap plastic centrifuge tubes containing tissues (5 g), homogenized at 20,000 rpm with a homogeniser probe for 1 min, and centrifuged for 10 min at 2500 g. A supernatant aliquot (2 mL) was washed once with hexane (2 mL); water (0.5 mL) was added and the mixture extracted twice with dichloromethane (2 mL). The combined dichloromethane layers were dried with anhydrous sodium sulfate and centrifuged. The whole supernatant was transferred to small test tubes and dried at 38°C under reduced pressure on RapidVap (Labconco, USA). The residue was resuspended in 80% aqueous methanol (0.5 mL) and transferred to autosampler vials. Aliquots (2.5 μL) were injected into the LC-MS system. For azaspiracids, edible tissue (5 g) was extracted with 90% aqueous methanol solution (20 mL) and treated as above.

### 3.2. Liquid chromatography-mass spectrometry analysis

LC-MS was performed on a Hewlett-Packard (HP) Model 1100 equipped with an in-line degasser, quaternary pump, autosampler and oven and coupled with an HP model 1100 Series single quadrupole mass spectrometer, through an atmospheric pressure ESI interface operated in the negative ion mode. Chromatograph operation, data collection and treatment were performed by HP *Chemstation 6* software. All LC-MS chromatograms presented were redrawn on Sigma Plot 4.0. Separation was achieved on a Merck Lichospher-100 RP-18 (5 μm, 125 × 2 mm I.D) column, protected by a guard column (4 × 4 mm I.D), also packed with Lichospher-100 RP-18 (5 μm). Column temperature was kept at 30°C. Mobile phase consisted of acetonitrile-0.05% acetic acid (65:35, v/v). Acetonitrile was of HPLC-grade and ultra-pure water was obtained on a Milli-Q system. Flow rate was set at 200μL/min, and analysis time at 9 min. After a 2-min separation the LC flow was introduced into the ESI interface without any splitting. The spray capillary voltage on the ESI interface was maintained at 4kV and the nebulizer pressure at 25 psig. High-purity nitrogen (obtained with a Whatman/HP N_2_-Generator) was used as a drying gas at 8.5 L/min. and 35°C. The fragmentor was kept at 180 V. Selected ion monitoring (SIM) was used to record the signals from the [M+H]^+^ ion at m/z 828.5 (AZA3), 842.5 (AZA1), 844.5 (AZA4, AZA5), 856.5 (AZA2) [15, 16]. For AZA’s calibration, contaminated Irish mussels with AZA1-5 from the Irish Marine Institute were used [7].

### 3.3. Enzyme-linked immunosorbent assays

The commercial “DSP-Check” kit, presently distributed by R-Biopharm, was employed for ELISA analysis. Extracts of edible tissues prepared for HPLC with 80% aqueous methanol were employed according to the kit instructions. Dilution of extracts with an equal amount of water led to a final concentration of aqueous 40% MeOH (Vale and Sampayo, [3,5]). Further dilutions were carried out in order for the toxin concentration to fall within the linear range of the test (0.01–0.1 μg/mL). The Kit comes only with two OA standard solutions: 10 and 100 ng/mL that are adequate for an eye-reading or semi-quantitative estimate.

For detecting all DSP toxin forms as in the HPLC assays above, the same hydrolyzed semi purified dichloromethane extract were used. Aliquots were dried in duplicate test tubes for HPLC and ELISA. As above, final dried residues contained 160 mg of edible mussel tissue. The test strips were read at 450 nm on an Elx-808 (Bio-Tek instruments, USA) microplate reader. The reader was controlled via KC4 (version 2.0, Bio-Tek) software, the 4-parameter logistic fits were done on Biograph (version 2.0, Bio-Tek). Results above or below the linear interval of the 4 parameter curve were not used for quantitative comparisons and classified as below or above the value that corresponded to the extreme values of linearity recommended by the kit instructions (0.01–0.1 μg OA/mL, respectively), multiplied by corresponding dilution plated. Due to the high cost of the commercial kit, only shellfish extracts whose DSP toxins had been previously detected (by HPLC) were used for ELISA assays. Shellfish analyses reported here are from those harvested in the Oualidia lagoon used for the 2006 monitoring.

## Figures and Tables

**Table 1.** Comparison of the results obtained by ELISA with those obtained from LC-MS for Mussels from Oualidia Lagoon harvested between May and August 2006 (values are in μg/100 g edible tissue). ELISA assays were performed according to the commercial “DSP-Check” kit instructions.

**Sampling Date**	**ELISA**	**LC-MS**		
		**Total (μg/100g)**	**OA μg/100g**	**DTX2 μg/100g**
02 May	12	3.5	3.5	0.0
15 May	4	2.7	2.7	0.0
22 May	21	20.6	20.6	0.0
29 May	29	19.1	19.1	0.0
05 June	42	38.0	38.0	0.0
12 June	113	134.6	134.6	0.0
26 June	81	92.4	92.4	0.0
03 July	68	68.6	62.5	6.0
10 July	50	77.7	60.1	17.5
17 July	13	36.7	27.3	9.4
24 July	13	32.2	20.9	11.3
15 August	4	11.5	11.5	0.0
22 August	3	8.8	8.8	0.0
